# Endoscopic endonasal approach for resection of odontoid process, decompression of the cervicomedullary junction spinal cord, and resection of pannus

**DOI:** 10.3171/2024.1.FOCVID23176

**Published:** 2024-04-01

**Authors:** David T. Fernandes Cabral, Ricardo J. Fernández-de Thomas, Ali Alattar, David A. Paul, Eric W. Wang, Paul A. Gardner

**Affiliations:** 1 Department of Neurological Surgery and; 2 Department of Otolaryngology, University of Pittsburgh Medical Center, Pittsburgh, Pennsylvania

**Keywords:** cervical myelopathy, craniocervical junction, endonasal, endoscopy, odontoid, odontoidectomy, pannus

## Abstract

Odontoid pannus is an abnormal collection of degenerative or inflammatory tissue on the C1-dens joint that can result in severe spinal cord compression myelopathy. Treatment options vary depending on severity and etiology. In cases of severe cord compression, surgical management could be either through a purely posterior approach or in combination with an anterior decompression via endoscopic endonasal approach (EEA). This case presents a 77-year-old female who underwent posterior cervical fixation for odontoid pannus causing dramatic cervical myelopathy who failed to improve over a 6-month period and required anterior transodontoid pannus resection and decompression via EEA.

The video can be found here: https://stream.cadmore.media/r10.3171/2024.1.FOCVID23176

## Transcript

We are presenting a case of an endoscopic endonasal approach for odontoidectomy, resection of an odontoid pannus, and anterior cervicomedullary junction decompression of the spinal cord.

### 0:32 Case Presentation.

The patient is a 77-year-old female with a history of spinal stenosis with progressive myelopathy. She presented to our institution with 1 year of progressive hand and feet numbness and tingling. She also had worsening of symptoms and weakness, particularly in the right upper extremity.

She initially presented to an outside hospital where she was found to have a large pannus at C1–2.^2^ Over there, they performed a posterior cervical fusion from C1 to C4, as well as C1–2 laminectomies.^1^ However, the patient failed to improve after this; in fact, she complained her symptoms were worsening. Of note, she also had a prior C4–7 ACDF that was done in 2019 to treat cervical radiculopathy.

### 1:18 Physical Exam.

Her physical exam was significant for bilateral upper-extremity weakness, right side worse than the left side. She was able to walk by herself; however, she had some difficulties with her balance.

### 1:30 Imaging Findings.

Her preoperative MRI at our hospital showed significant compression of the spinal cord from an anterior vector coming from this odontoid pannus, as we can see here in the sagittal and axial images. We can also notice that she also has T2 changes already in her spinal cord.

Her preoperative CT shows the hardware from the C1–4 posterior cervical fusion as well as her prior anterior cervical decompression and fusion.

We also obtained a preoperative CTA that we obtain routinely in our patients to evaluate the location of the bilateral internal carotid arteries. In this particular case, you can see the intercarotid distance is narrow, which is a factor to consider when performing the rhinopharyngeal flap, as we are going to discuss later.

### 2:25 Positioning.

The patient is positioned supine in the operating table with a left shoulder bump to have the patient facing toward us. The head is fixed in a 3-pin Mayfield in neutral position to avoid C1 from rotating on top of C2, which could bring one of the lateral masses anteriorly, difficulting the resection of the anterior arch of C1 and the odontoid.^3,4^

### 2:47 Surgical Indication.

We decided to proceed with an odontoidectomy since the patient’s symptoms actually got worse after 6 months from her prior surgery.

### 2:57 Surgical Trajectory.

Here we can see the surgical corridor, and also we can see the inferior extent to the limit of our resection of the odontoid, which is delineated by the nasopalatine line, which has been described in prior publications by our team. This line has been represented here by the yellow arrow, and the area that can be resected from the odontoid has been represented in red color.

### 3:21 Nasal Approach.

We perform the entire surgery in collaboration with the ENT team. They start injecting some epinephrine on the mucosa of the nasal septum on each side. They also perform lateralization of the inferior turbinate in both sides. This allows to have more space to pass the instruments through the nose.

### 3:41 Posterior Septectomy.

We then perform a posterior septectomy with a needle-tip electrocautery. These cuts are done at the level of the choana, and bring anteriorly for about an inch, and then is turned inferiorly at the junction of the septum with the nasal floor. The second cut with the needle-tip Bovie is done at the junction of the septum and the nasal floor. This is done bilaterally. Once the mucosa is cut, it is subsequently elevated with a Cottle dissector. It is really important to notice here that this septectomy is done posterior and inferior to avoid damage to the nasal flap pedicle. We then perform two cuts with a back-biter in the posterior aspect of the septum to release the mucosa, which is then removed. The exposed bone from the septum is then fractured with a Cottle dissector. We then remove any bone spicules to have a smooth surface to work.

### 4:40 Maxillary Crest Drilling.

We drill the maxillary crest with a 4-mm diamond burr. This creates a smooth surface to work and facilitate the binostril access of this area, as well as allows us to reach down to the area of interest.

### 4:54 Corroboration of C1–2 and Internal Carotid Arteries Location.

We use navigation to confirm that we can reach below the anterior arch of C1. We also use Doppler and image guidance to study the location of the ICAs bilaterally.

### 5:07 Rhinopharyngeal Flap Dissection.

Once we know where the internal carotid arteries are, we then start performing our cut with the needle-tip cautery. We started in this case in the left side, and then we are going to perform the superior cut of the rhinopharyngeal flap. In this particular case, because the carotid arteries were close to each other, meaning that the intercarotid distance was narrow, we decided it was safer to a vertical split of the rhinopharyngeal flap in a "T" shape instead of an inverted "U" shape. The dissection of the rhinopharyngeal flap is done very carefully; we dissect this flap off the anterior surface of the inferior clivus all the way down to the vertebral body of C2, and it is carried out as lateral as safe as possible.

### 5:59 Identification and Localization of the Lateral Masses of C1 and the Anterior Tubercle of C1.

We use navigation to confirm the location of the lateral masses of C1. We can also see here very clearly the anterior tubercle of C1, which is a midline structure.

### 6:11 Drilling of the Anterior Arch of C1.

A high-speed drill was then used to remove the anterior ring of C1 between both lateral masses. After this drilling is completed, with the help of a Kerrison rongeur we disconnect the soft tissue off the anterior ring of C1, and this part of the bone is then removed, exposing the odontoid process of C2.

### 6:38 Retraction of the Rhinopharyngeal Flaps.

We then used a 4-0 Vicryl stitch non–pop-up to retract each side of the rhinopharyngeal flap as you can see here. The thread from each stitch is pulled out of the nose through the respective nostril, and then it is secured under tension to the patient’s drape with a mosquito.

### 7:03 Initial Pannus Resection and Odontoidectomy.

We then remove part of the pannus that is anterior to the odontoid process and we start drilling the dens from top to bottom to keep it from disconnecting from the vertebral body of C2, which would create a tip of the dens that is floating with significant ligamentous attachments. We continue to drill the dens all the way down to the vertebral body of C2. This drilling is also carried out posteriorly until a very thin rim of C2 dens is left behind. We then remove this posterior cortex of the odontoid process with the help of a Kerrison rongeur. You can see here all this pannus that is posterior to the dens, which is the area that it is compressing the spinal cord.

### 7:52 Pannus Resection.

We use a combination of Kerrison rongeur, as well as pituitary and ethmoidals, to resect this pannus off the dura. We resect all this abnormal tissue very carefully off the dura to avoid causing a durotomy.

### 8:19 Final Pannus Resection and Dura Inspection.

We continue to remove this pannus all the way deep to the level of the dura until the dura is free, is ballooning, and pulsatile, as we can appreciate on this segment of the video.

### 8:34 Hemostasis.

Hemostasis is easily achieved with a combination of bipolar electrocautery, Surgifoam, and warm irrigation.

### 8:43 Retention Stitches Removal.

We then remove the stitches that were holding on each side the RP flap.

### 8:50 Rhinopharyngeal Flap Suturing.

We use a 4-0 V-Loc stitch to put together each piece of the rhinopharyngeal flap. Once the RP flap has been sutured, we then proceed to cut the stitch and then we keep the flap in place with the help of a Merocel packing that it is placed by our ENT colleagues.

### 9:22 Surgery Length.

The surgery duration was 3 hours and the blood loss was 200 cc.

### 9:26 Postoperative Course.

The patient tolerated the procedure well and spent 1 night in the ICU. She subsequently was discharged to inpatient rehab where she spent 1 week and then she went home.

Her pathology report confirmed an odontoid pannus with fibroconnective tissue.

### 9:41 Postoperative Exam.

Her upper-extremity strength improved as well as her gait postoperatively.

### 9:47 Postoperative Images.

Her postoperative CT shows complete resection of the odontoid process and in the axial sequences you can see the removal of the anterior ring of C1. Her MRI shows adequate decompression of the ventral aspect of the spinal cord at the cranio-cervical junction. This can be also seen here in this comparison of her preoperative and her postoperative images.

## Disclosures

The authors report no conflict of interest concerning the materials or methods used in this study or the findings specified in this publication.

## Author Contributions

Primary surgeon: Gardner. Assistant surgeon: Fernandes Cabral, Alattar, Wang. Editing and drafting the video and abstract: Gardner, Fernandes Cabral, Fernández-de Thomas, Paul. Critically revising the work: Gardner, Fernandes Cabral, Fernández-de Thomas, Alattar, Wang. Reviewed submitted version of the work: all authors. Approved the final version of the work on behalf of all authors: Gardner. Supervision: Wang.

## Supplemental Information

### Patient Informed Consent

The necessary patient informed consent was obtained in this study.
